# Exploring the Impact of the Obesity Paradox on Lung Cancer and Other Malignancies

**DOI:** 10.3390/cancers14061440

**Published:** 2022-03-11

**Authors:** Lindsay Joyce Nitsche, Sarbajit Mukherjee, Kareena Cheruvu, Cathleen Krabak, Rohit Rachala, Kalyan Ratnakaram, Priyanka Sharma, Maddy Singh, Sai Yendamuri

**Affiliations:** 1 Department of Thoracic Surgery, Roswell Park Comprehensive Cancer Center, Elm and Carlton Streets, Buffalo, NY 14263, USA; ljnitsch@buffalo.edu (L.J.N.); kareenacheruvu@gmail.com (K.C.); cakrabak@davidson.edu (C.K.); rachala.rohit@nicholsschool.org (R.R.); kkratna13@gmail.com (K.R.); priyankasharma562@yahoo.com (P.S.); maddy.singh@gmail.com (M.S.); 2 Department of Medicine, Roswell Park Comprehensive Cancer Center, Elm and Carlton Streets, Buffalo, NY 14263, USA; sarbajit.mukherjee@roswellpark.org

**Keywords:** obesity, BMI, lung cancer, obesity paradox

## Abstract

**Simple Summary:**

Studies have shown that obesity is associated with many adverse health effects, including worse cancer outcomes. Many studies paradoxically suggest a survival benefit for obesity in treatment outcomes of cancers such as non-small-cell lung cancer. This relationship is not seen in animal models. We hypothesize that this relationship is secondary to suboptimal quantification of adiposity, enhanced immunotherapy response, and variables such as sex, medications, and smoking status. There are many ways to measure and classify adiposity, but the ability to distinguish abdominal obesity is likely key in predicting accurate prognosis. There are many ways obesity impacts cancer treatment course from diagnosis to survivorship. In this paper, we aim to analyze the factors contributing to the obesity paradox and its effect on lung cancer. This can aid the treatment and prognosis of lung cancer and may support further research into obesity-specific impacts on this malignancy.

**Abstract:**

There is a paradoxical relationship between obesity, as measured by BMI, and many types of cancer, including non-small-cell lung cancer. Obese non-small-cell lung cancer patients have been shown to fare better than their non-obese counterparts. To analyze the multifaceted effects of obesity on oncologic outcomes, we reviewed the literature on the obesity paradox, methods to measure adiposity, the obesity-related derangements in immunology and metabolism, and the oncologic impact of confounding variables such as gender, smoking, and concomitant medications such as statins and metformin. We analyzed how these aspects may contribute to the obesity paradox and cancer outcomes with a focus on lung cancer. We concluded that the use of BMI to measure adiposity is limited and should be replaced by a method that can differentiate abdominal obesity. We also concluded that the concomitant metabolic and immunologic derangements caused by obesity contribute to the obesity paradox. Medications, gender, and smoking are additional variables that impact oncologic outcomes, and further research needs to be performed to solidify the mechanisms.

## 1. Introduction

It has been established that obesity (BMI > 30 kg/m^2^) is a major risk factor for many types of cancer, accounting for approximately 20% of all cancer cases over the past 25 years [1]. This is particularly concerning considering that the proportion of the population that is obese continues to climb [2]. Increased body mass index (BMI) has been shown to increase the development of multiple malignancies [3] and worsen their prognosis. Excess adiposity has been hypothesized to act as a carcinogen by increasing inflammation, metastatic potential, angiogenesis, and evasion of apoptosis [4]. Obesity promotes upregulation of growth-inducing hormones and increases insulin-related growth factors such as IGF-1 [4]. In addition to the increase in the mortality rate, obese patients have worse quality of life following cancer treatment [5].

Although obesity is perspicaciously an adverse prognostic factor in many disease states [6,7,8,9], a nuanced relationship exists between obesity and lung cancer outcomes, termed the obesity paradox [10,11,12,13,14]. This relationship has been shown in early- [15] and late-stage [12,13] non-small-cell lung cancer (NSCLC). This relationship is consistent enough that obesity has been listed as a negative predictive factor for the development of lung cancer in algorithms [16].

The obesity paradox has motivated researchers to analyze further how clinicians measure and quantify obesity and contemplate the limitations of BMI. Although conventional and convenient, BMI may be suboptimal and a major contributing factor to the obesity paradox because it cannot distinguish abdominal obesity [17]. Researchers have also explored immunologic derangements as a contributing factor to the obesity paradox [18]. It has been proposed that obese patients respond better to immunologic therapies such as pembrolizumab, which is utilized in NSCLC, since they over-express PD-1 on T cells [16]. It has been shown that obesity-related inflammation contributes to increased myeloid-derived suppressor cells (MDSCs) [19,20]. Metformin and smoking are additional confounding variables in the obesity paradox due to their disproportional use in the obese and non-obese populations, respectively [21]. The rationale for completing this study is the continued debate and confusion surrounding the impact of obesity on long-term NSCLC outcomes. This study integrates recent studies that investigate the impact of visceral adiposity and particularly central adiposity. This study also postulates possible contributions of obesity-associated medications such as metformin and statins. Additionally, this study supports the use of imaging densitometry analysis rather than a continued reliance on BMI for quantifying body habitus.

## 2. Materials and Methods

We utilized computerized databases to identify literature on obesity and its effect on oncologic outcomes. Specifically, we analyzed published literature pertaining to the diagnostic methods utilized to quantify adiposity, metabolic derangements caused by obesity, the effect of metformin and statins on oncologic outcomes, the impact of the obesity paradox on NSCLC as well as other cancers, immunologic derangements resulting from obesity, and confounding factors such as sex and smoking status. We performed a literature search using PubMed from March 2021 to September 2021, analyzing articles published between 1980 and 2021. The search terms used included obesity paradox, lung cancer, cancer, central adiposity, visceral adiposity, lung cancer long-term survival, and immunotherapy. Articles were included for consideration if they were published within the specified time frame, were original studies or a meta-analysis of original studies and pertained to the impact of obesity on: cancer survival, pathogenesis, and prognosis and immunology. Exclusion criteria for articles included out of range publication date, repeated articles, and articles that preceded a subsequent larger study performed by the authors. At least three researchers read each article independently to ensure relevance and confirm the quality of the source. The principal author settled all discrepancies. By using a large database to collect literature and the fact that the articles were read by multiple investigators independently, the risk for bias was reduced.

After deciding to include a study, the authors sorted the papers according to their subject matter in order to formulate a thorough encapsulation of the data. As an example, all of the papers pertaining to the impact of obesity on tumor pathogenesis were read together prior to writing that section of this paper. The manuscripts were then qualitatively weighed based on the strength of the evidence they included. This was then incorporated into a coherent narrative.

## 3. Results

### 3.1. Obesity and Cancer Development

Obesity is an epidemiologic problem that will continue to grow in the coming years. CDC data reported that 42.4% of American adults were obese in 2018, and that number increased from 30.5% in 2000 [22]. As well as the negative public health care impacts, obesity is a significant strain on health care spending, with 147 billion dollars spent in 2008 [22]. Much of this spending is secondary to the diseases often concomitant with obesity, such as heart disease [23], type 2 diabetes [24], hypertension [25], asthma [26,27], and cancers [28,29]. When the costs of inpatient care, outpatient care, emergency care, dental care, and prescriptions were analyzed, it was found that obesity (BMI > 30) accounted for 149.4 billion dollars in 2014 [28].

An early association between obesity and cancer risk was made in 1966 by analyzing trends in endometrial cancer [30]. The authors proposed increased estrogen exposure as the pathogenesis of the heightened oncologic risk in obese women [30]. Since that time, many groups have shown associations between increased BMI and the development of cancer. Obesity is a major cause of the non-alcoholic fatty liver disease (NAFLD), a leading cause of hepatocellular carcinoma (HCC) [31,32,33]. Obesity increases the risk of non-Hodgkin lymphomas [34,35], particularly diffuse large B-cell lymphomas (DLBCL) [36]. Obesity showed a significant increase in the incidence of gastric cardia cancer [37], thyroid cancer [38], colon cancer [39], renal cancer [40,41,42], liver cancer [43], malignant melanoma [44], multiple myeloma [45], rectal cancer [39], gallbladder cancer [46], leukemia [47], esophageal cancer [37], and, as previously mentioned, non-Hodgkin lymphoma [48].

It has been suggested that childhood obesity contributes to increased cancer risk and cardiovascular disease risk in adulthood [49]. This is concerning because childhood obesity was pronounced an epidemic affecting 17% of the children [50] in the US. It is likely related to the fact that if a person is obese as a child, they also tend to be obese as an adult. Increased BMI as a teen was associated with a higher risk for leukemia (OR = 1.32, 95% CI 1.15–1.53) [51,52], Hodgkin’s disease (HR = 1.25, 95% CI 1.13–1.37) [49,53], and colon cancer (39% increase in men and 19% increase in women) [54].

The increase in cancer development has been proposed to be secondary to immunologic and metabolic derangements in the tumor microenvironment [55]. Obesity has been said to create a “meta-inflammatory” state through the body, leading to impaired immunologic recruitment, coordination, and response [56]. Obesity has been shown to contribute to increased cytokine production, aberrant macrophage activity, and increased exhaustion phenotypes shown by immune cells [57]. Obesity-associated inflammation contributes to increased myeloid-derived suppressors cells (MDSCs), which suppress innate and adaptive immune responses [19,20]. Usually, cells of myeloid lineage progress to become macrophages, granulocytes, and dendritic cells [58]. If these cells instead become MDSCs, they may inhibit natural killer cells and T-cell cytotoxicity and increase regulatory T cells by liberating factors such as arginase-1 and inducible nitric oxide synthase [59]. Natural killer cells defend the body against foreign matter and neoplasms by releasing cytoplasmic mediators or cytotoxic activity [60,61]. When the tumor microenvironment is altered so that there is an abundance of pro-inflammatory phenotypes and increased TGF-β, the natural killer cells are less protective against carcinogenesis [62]. In particular, it has been shown that the natural killer cells in an obese patient struggle to activate glycolysis [63], which is essential for activating their cytotoxic machinery. It was postulated that this is due to increased lipid uptake by the natural killer cells [63]. It has been shown that adipose tissue serves as a reservoir for cytokines, including TNF-α, leading to inflammation [64]. This effect is seen in animal models, with obese dogs exhibiting higher IL-6 and TNF-α levels and lower T-cell proliferation than healthy weight dogs [65]. It has been suggested that increased adipose tissue promotes that pivoting of a Th2 phenotype to a Th1 and Th17 predominant phenotype [66]. This shift and the increase in liberated cytokines contribute to a pro-inflammatory state [55].

Obesity increases the liberation of leptin and other adipokines due to an increase in adipose tissue volume [67]. Leptin has been shown to promote cancer growth in mice [67]. Obesity decreases adiponectin levels, an adipokine that counters the cancer-promoting effects of leptin [67]. In conjunction with increased leptin release, the downregulation of adiponectin allows increased growth and metastasis. Obesity alters levels of steroid hormones which can contribute to cancer growth [67]. Estrogen can be produced by adipose tissue [68]; in fact, it is the primary way estrogen is produced in postmenopausal women [67]. Estrogen has been shown to induce cancer-promoting effects, such as inhibiting apoptosis and inducing angiogenesis [67]. It has been shown that estrogen promotes tumor growth and angiogenesis in mice, even in cancer cells lacking the 17β-estradiol receptor [68]. Obesity also reduces the sequestration of estrogen by downregulating levels of sex hormone-binding globulin, leading to increased circulating unbound estrogen [69]. Obesity leads to the dysregulation of insulin signaling, negatively impacting cellular signaling pathways and promoting cancer growth. Obesity causes increased insulin release due to decreased insulin responsiveness [70]. In addition to its metabolic effects, insulin promotes mitosis [67]. Cancer cells exposed to insulin divide faster, leading to increased tumor growth [71]. Insulin signals through the PI3K and MAPK pathways are common to many crucial signaling cascades that regulate cellular functioning and metabolism [67]. Consequently, dysfunction in these pathways secondary to improper insulin signaling can lead to derangements in normal cell development and cancer [67]. Increased levels of IGF-2 (insulin-like growth factor 2) methylation are correlated with obesity and insulin resistance [72,73]. IGF-2 may bind to IR-A (insulin receptor A) or IGF1R (insulin-like growth factor 1 receptor) to increase glucose uptake and activate anabolic pathways, leading to energy production, cell growth, and division [67]. Furthermore, high-fat diet-induced obesity leads to a metabolic competition between tumor and CD8 T cells for lipids, leading to increased lipid uptake by tumor cells than T cells, thereby impairing CD8 T-cell infiltration and function [74].

### 3.2. Obesity and Cancer Prognosis

Along with increasing the incidence of cancers, obese patients tend to have compromised survival and increased complications following cancer treatment. Prostate cancer-specific mortality and biochemical recurrence were increased by 15% and 21%, respectively, with a 5 kg/m^2^ weight gain [75]. In colorectal cancer, obese patients have elevated all-cause mortality, cancer-specific mortality, disease recurrence, and decreased disease-free survival [76]. These negative prognostic factors were not seen when analyzing patients with BMIs characterized as overweight [76]. Obesity compromised survival and post-operative outcomes of recurrent hepatocellular carcinoma [77]. When operated on for hepatic metastasis of colorectal cancer, obese patients spent a longer time in the operating room and had higher estimated blood loss, morbidity, and reintervention rates when compared to non-obese peers [78]. A higher BMI was associated with extrathyroidal and vascular invasion of papillary thyroid carcinoma, suggesting obesity leads to a more aggressive phenotype [79]. Obesity was associated with increased wound dehiscence, incisional site hernia, and stoma complications following colorectal surgery, and these patients were at higher risk for conversion to an open surgery [80]. Obese patients were found to present with melanomas that were twice as thick as their non-obese peers [81]. Following surgical resection of squamous cell carcinoma (SCC) of the tongue, obese patients had worse disease-specific survival (DSS), recurrence-free survival (RFS), and overall survival (OS) [82]. Elderly female patients with B-cell lymphomas treated with RCHOP had a worse prognosis if they were obese [83]. Following resection of gastric cancer, patients with a BMI > 30 experienced increased hospital length of stay (LOS), OR time, post-operative morbidity, and post-operative mortality [84]. It was shown that the inflammation and desmoplasia caused by obesity led to a more aggressive phenotype of pancreatic adenocarcinoma that was resistant to chemotherapy and was prone to progression [85]. It was also found that patients with a BMI >35 kg/m^2^ were 12-fold more likely to have lymph node-positive disease and decreased DFS and OS [86]. Obesity leads to poor prognosis in patients undergoing surgical resection of extremity soft-tissue sarcoma resection. This is likely due to poor wound healing, increased dehiscence, and increased infections [87].

### 3.3. Obesity Paradox in Other Cancers

#### 3.3.1. Renal

It has been hypothesized that there is an obesity paradox in renal cell carcinoma (RCC) patients. RCC is the most common type of kidney cancer and is 2.4% of all adult cancers [88]. Adiposity is a significant risk factor for developing RCC but may improve prognosis [88]. The increased incidence is thought to be secondary to insulin resistance and the increase in IGF-1. This elevated IGF-1 suppresses Bcl-2 to decrease apoptosis while simultaneously increasing proliferative and angiogenic factors [89]. Possible explanations for the improved prognosis echo many of the same sentiments cited in lung cancer, namely the use of BMI for patient categorization and alterations of the tumor immune microenvironment, with obese patients exhibiting higher levels of IL-6 TNF-α and c-peptide [90]. Multiple studies have shown an increase in overall survival and progression-free survival in obese patients [91]. This effect was seen in patients with metastatic disease [92] and those who underwent nephrectomy [93,94]. Similar to how obese patients respond better to PD-1 inhibitor therapy, obese RCC patients tend to respond better and have longer OS when treated with anti-VEGF therapies such as sunitinib, sorafenib, and bevacizumab, and axitinib [95,96]. Therefore, there is an apparent obesity paradox seen in RCC when patients are treated with immunotherapy.

#### 3.3.2. Melanoma

Similar to NSCLC and RCC, obese melanoma patients treated with immunotherapy tended to have increased survival and better checkpoint inhibitor response than their normal-weight peers [97]. Obese patients were observed to respond more robustly to immune checkpoint inhibitors and have increased OS [98]. Significantly better reactions to dabrafenib, ipilimumab, trametinib, the BRAF inhibitor vemurafenib, and PD-1 therapies have been observed in obese patients [99,100]. Similar to the mice experiments performed in lung cancer, obese mice with melanoma treated with PD-1 therapy showed a better response than normal-weight mice [101] Therefore, there is an apparent obesity paradox in melanoma patients that becomes apparent when they are treated with immunotherapy.

### 3.4. Obesity Paradox in Lung Cancer

#### 3.4.1. Obesity and Survival in Lung Cancer

The notion that obesity may be protective against lung cancer or contribute to improved outcomes has been a paradoxical clinical conundrum. Part of this controversy involves its absolute deviation from data obtained in animal experiments which show obesity is negatively associated with NSCLC survival [102,103]. It was thought that when undergoing surgery for lung cancer, obese patients would experience more complications. This was thought to be secondary to having less excursion of the diaphragm, lower lung volumes due to restriction, and relative immobility, but this has been disproven [104]. A relationship was found between being underweight and compromised post-operative outcomes, with these patients experiencing more surgical and infectious complications [105,106]. It has also been shown that in-hospital morbidity and mortality are significantly decreased in obese patients and can be a predictor for long-term survival [107] (Table 1). Obesity improves prognosis in operable NSCLC as shown by an increased OS [108,109,110]. When mortality in the years following surgical resection of locally advanced lung cancer was analyzed, obesity confirmed a survival benefit [13]. In a large metanalysis of 76,086 patients, obesity increased lung cancer survival [111]. This effect was seen in patients receiving carboplatin-paclitaxel chemotherapy, with obese patients experiencing no decreased survival or increased toxicity [112]. When 676 patients were treated with docetaxel in the OAK and POPLAR trials, BMI did not affect overall survival (HR = 0.96, 95% CI 0.78–1.18) for overweight and (HR = 0.92, CI 95% 0.70–1.21) for obese patients [113]. When patients with a BMI of 18.5–25 where compared to patients with a BMI above 25, the higher-BMI patients had better overall survival (HR = 1.42 95% CI 1.14–1.78) [114]. When a BMI of 25 kg/m^2^ was used as a cutoff, higher-BMI patients were at a 5.3-fold increased risk of post-operative respiratory complications [115] (Table 1).

#### 3.4.2. Enhanced PD-1 Checkpoint Inhibitor Therapy Response

PD-1 is present on all activated T cells and binds to PD-L1 [116]. When tumor cells express PD-L1, the local T cells in the microenvironment become exhausted, dysfunctional, and neutralized and can no longer contribute to host defense [116]. PD-1 inhibitors include monoclonal antibodies such as pembrolizumab and nivolumab that target the PD-1 receptor on T cells to release the negative regulator control [117]. This is typically in place to allow for anergy and mitigate autoimmune insults. By removing this control, the goal is to increase neoplasm destruction [117]. These medications have also been beneficial to reactivate and recruit cytotoxic T cells [117].

It has been shown that patients with a BMI > 25 (kg/m^2^) had a significantly better overall survival [118] and a better immunologic response [16,113] following treatment with a PD-1 inhibitor, pembrolizumab. This phenomenon was also seen in melanoma [98,100] and renal cell carcinoma patients [118,119].

In animal experiments, diet-induced obesity (DIO) mice treated with a PD-1 inhibitor experienced less B16-F0 melanoma and 3LL lung carcinoma tumor growth (expressed as lower tumor volume) and increased tumor PD-1 inhibitor response (shown by decreasing tumor size) compared to their average weight counter-parts. [101]. The DIO mice had less tumor growth at days 11 and 16 [101], and this effect was additionally seen when DIO mice were inoculated with lung tumors. The DIO mice had higher T-cell infiltration and increased CD8 to CD4 ratio [101]. There were also fewer PDL1+ T cells in the TME, proving that T cells were rescued from an exhausted state [120]. Increased expression of PD-1 may be why obese mice and people fare better [120]. This increase may be secondary to an increase in leptin [101], often elevated in obesity [121], leading to increased CD8^+^ T cells and STAT3, which is both a signal transducer and activator of transcription [122]. The increased expression of STAT leads to a higher PD-1 expression [101] and the phenotype of T-cell exhaustion seen in obese patients [101]. This survival advantage for obese patients is not seen when treated with conventional chemotherapy such as docetaxel, suggesting that the survival advantage is due to better PD-1 checkpoint inhibitor response [123,124].

### 3.5. Measurements of Adiposity

#### 3.5.1. BMI

Body mass index (BMI) is the most common form of measurement for obesity [125]. To calculate BMI, one must divide weight in kilograms by height in meters squared. Obesity is further stratified into risk groups.

Some advantages of using BMI to measure obesity are that it is inexpensive, allows risk stratification, is strongly correlated with body fat levels, and is easy to measure [125]. BMI is not a reliable measurement of obesity because it measures total body mass, not only adipose tissue [17], and fails to identify abdominal obesity. This impacts patients because some adiposity patterns predispose them to more adverse sequelae than others. For example, increased visceral fat carries a worse prognosis than increased subcutaneous fat [126]. In a study examining the effect of obesity on lung cancer risk, BMI was inversely associated with the risk of lung cancer, but two other measures of obesity, the waist-to-hip ratio (WHR) and A Body Size Index (ABSI), showed positive correlations with lung cancer risk [127].

#### 3.5.2. Underwater Weighing

To measure body habitus using underwater weighing, individuals are weighed in air and submerged in a tank with an underwater seat hanging from a scale [128]. Researchers then calculate body volume, body density, and body fat percentage due to the difference in densities [128]. Underwater weighing is accurate but is inaccessible to children, older adults, people with a BMI of 40+, and health-related problems due to the requirement of complete submersion [128].

#### 3.5.3. Dual-Energy X-ray Absorptiometry

Dual-energy X-ray absorptiometry uses two low-level X-ray beams to estimate fat-free mass, fat mass, and bone mineral density [128]. Though incredibly accurate, the method is expensive and cumbersome and is suboptimal for patients with a BMI > 35 kg/m [2] and those who are pregnant due to radiation exposure [128].

#### 3.5.4. Bioelectric Impedance

Bioelectric impedance utilizes the differences in resistance between tissue types when bombarded with electric current to estimate body water from which body fat is computed [129]. The method is convenient, safe, relatively inexpensive, and fast [129]. Limitations include device calibration and the inaccuracy introduced during illness, dehydration, weight loss, and a BMI of approximately 35 or higher [129].

#### 3.5.5. The Waist-to-Hip Ratio

The waist-to-hip ratio measures obesity by dividing the waist measurement (cm) by the hip measurement (cm) [130]. Like BMI, it is inexpensive and convenient but is prone to measurement error and is less accurate to measure in individuals with a BMI of 35 kg/m^2^ or greater [130]. Turning the measured data into a ratio leads to a loss of information mathematically; two people with very different BMIs can have the same waist-to-hip ratio.

Although the waist-to-hip ratio and waist circumference are easy to measure and validate, a detriment to these methods is that they need to be performed prospectively and add additional burden on the patient [18]. There may be additional challenges getting subsequent measurements, and patients may be lost to follow up or pass away before the data can be collected [18]. The waist-to-hip ratio has the advantage of detecting abdominal obesity, which may be a better predictor of the development of lung cancer [131].

#### 3.5.6. Visceral Fat Index

Visceral fat index (VFI) utilizes densitometry analysis of the routine computer tomography (CT) scans at the level of L3 to quantify visceral and subcutaneous adiposity [18]. The visceral adiposity area is then normalized to the patient’s height to allow comparative analysis [18]. This method can be performed retrospectively, does not require additional work by the patient, and mitigates many of the current qualms pertaining to BMI [18]. An additional advantage of this method is that the scans are frozen when taken, so we can accurately analyze patients retrospectively, unlike the other methods. It also allows for more granulated data that can be subsequently analyzed, and higher-level conclusions may be drawn about the impact of adiposity distributions [18]. This method has been shown to highly correlate with volumetric determinations of subcutaneous and visceral adipose tissue [132]. By utilizing VFI, we have been able to definitively show that increased visceral fat compromises overall survival and recurrence-free survival in NSCLC [18]. We were able to show that visceral obesity, as defined by a relatively high VFI, was inversely associated with inflammatory genes within the tumor microenvironment [18]. This suggests that visceral adiposity may downregulate the anti-tumor immune response and lead to progression and advanced disease [18].

#### 3.5.7. Abdominal Obesity

Abdominal obesity is a known risk factor for both cardiovascular disease and the development of many cancers [133,134,135]. The age-adjusted prevalence of abdominal obesity increased from 46.4% of adults in 1999–2000 to 54.2% in 2011–2012, and this trend is consistent regardless of gender and ethnicity [136]. This increase occurred independently of an increase in the average BMI as seen by the increase in the average waist circumference by 0.2 cm in men and 2.4 cm in women in the same time span [136]. It has been shown that for each 0.1 unit increase in the waist-to-hip ratio, there was a 5% increased rate of developing lung cancer [131]. Abdominal obesity has more clearly been associated with an increased risk of NSCLC than general obesity [137,138,139]. This association’s proposed mechanisms include hyperinsulinemia, increased unbound androgen level, increased estrogens, and lower levels of sex hormone-binding globulin [139,140,141,142,143]. All of the characteristics above are explicitly seen in association with abdominal obesity. Abdominal obesity also predisposes patients to the development of insulin-resistant diabetes, contributing to inflammatory and immune sequala [136].

### 3.6. Confounding Factors

#### 3.6.1. Metformin

It has been shown that type 2 diabetes is associated with a higher risk of developing lung cancer [144,145]. When analyzing the obesity paradox, a major confounding factor is the use of the anti-diabetic agent metformin. It was found that 52% of patients with type 2 diabetes mellitus were found to be obese (BMI > 30 kg/m^2^), and 8.1% of patients were found to be morbidly obese (BMI > 40 kg/m^2^) [146]. Commonly used in type 2 diabetes, metformin has been associated with decreased cancer development [147]. A major protective mechanism of metformin is decreasing the development of tobacco-associated cancer development [21]. It has been shown that the protective effects of metformin are disproportionately seen in obese or overweight patients [148,149].

Women diagnosed with early-stage breast cancer taking metformin had 22% lower fasting insulin levels and had several improved metabolic factors such as lower total cholesterol [150]. These metabolic benefits also extended to patients with metabolic syndrome secondary to androgen deprivation therapy for prostate cancer [151]. In addition to its metabolic benefits, metformin has also been shown to inhibit cancer cell proliferation; therefore, it may have utility as an adjuvant therapy [151].

A study investigating the impact of metformin on chemotherapy patients found that its anti-tumor behavior is secondary to the activation of adenosine monophosphate kinase (AMPK), the modulation of adenosine A1 receptors, a reduction in insulin/insulin growth factors, and its role in blocking endogenous reactive oxygen species [152].

Metformin appears to decrease the proliferation and growth of certain forms of cancer in preclinical studies [153,154]. Metformin achieves these effects by decreasing the biosynthetic activity of mitochondria by decreasing Krebs cycle activity and lipid synthesis, effectively starving it for nutrients [154]. Given the potential for differences in metformin responses in cancer patients, its therapeutic uses may be limited to target populations, and advantageous genetic polymorphisms remain unknown [155]. Metformin’s efficacy as neoadjuvant therapy in cancer patients has yet to be proven [155].

#### 3.6.2. Statins

The statin class of medications treats high cholesterol and works by blocking HMG-CoA reductase. Statins lower LDL and raise HDL. Advantages of these medications include a reduction in atherosclerosis lower coronary artery disease [156]. They can be used for primary or secondary prevention [156]. Similar to how metformin is more likely to be on the medication list of a patient who has diabetes, statins are more likely to be used by those with hypercholesteremia. Both diabetes and high cholesterol are associated with obesity.

Statins have improved overall survival in stage four esophageal cancer patients [157]. Other studies showed improved outcomes in statin users when used after diagnosis [158,159]. Our group was able to show an overall and cancer-specific survival benefit of statins when they were used prior to esophageal adenocarcinoma diagnosis [157].

This benefit has also been seen in stage IV lung cancer [160]. In patients who are overweight or obese, statin use was associated with improved recurrence-free survival [161]. These higher-BMI patients who used statin were found to have higher expression of granzyme A and interferon-γ, which are tumoricidal [161].

The likely mechanism of this survival advantage is the pro-apoptotic and anti-proliferative effects caused by the statin medications [157]. Multiple mechanisms have been proposed, including endoplasmic reticulum stress, caspase induction, NF-kB blockade, and mTOR inhibition [157].

#### 3.6.3. Sex

It has been shown that there are sex-specific differences in adipocyte storage and tissue metabolism [162]. Biological males tend to experience android adipose deposition, with concentrations in the upper body and abdominal areas [162]. Biological females tend to deposit adipose in a gynoid pattern, with higher concentrations in their lower bodies. The differences in deposition are thought to be due to the relative differences in concentration of sex hormones which also explains the reason for these changes to be more pronounced prior to menopause [163]. An additional explanation that has been shown in mice hypothesizes that the changes may be due to the sex chromosomes themselves [164]. In mice, it was observed that mice with XYY had increased visceral adiposity [165].

In addition to affecting the way adiposity deposits, recent data have suggested that there may be a differential response to immunotherapy due to a patient’s sex [166]. This has been explained by the differences in sex hormone levels and genes whose expression is based on sex chromosomes [167]. Females often mount more aggressive immune responses than men, as displayed by the much higher rate of autoimmune diseases in females than men [168,169]. When immunotherapy is used alone, men often fair better than women [170]. These results have been seen in PD-1 and CTLA-4 inhibitors [171,172].

In male NSCLC patients, immunotherapy using anti-PDL1 inhibitors vs. chemotherapy showed improved outcomes while the same effect was not seen in female patients [173]. The trend of men benefitting more from immunotherapy was supported by a 2018 analysis of RCTs, with men showing significantly improved survival and efficacy when compared to women [174]. It was found that when metastatic melanoma patients were treated with immunotherapy, those who had the best long-term survival were male patients who were overweight or obese compared to average BMI patients and women who were overweight and obese [100].

Interestingly, when combined with chemotherapy in advanced lung cancer, female patients had larger benefits from immune checkpoint inhibitors than men. A possible mechanism is that chemotherapy influences the tumor to accumulate more mutations to survive, and therefore the immunotherapy has more targets to attack in an already strong female immune system [166]. Another potential explanation for these differences could be the sex-associated differences in the gut microbiota composition [175]. Finally, male-derived tumors often have higher tumor mutational burden (TMB) and cancer germline antigen expression than those from females, and therefore males may benefit more from immunotherapy [160].

#### 3.6.4. Smoking

It has been extensively proven that smoking dramatically increases the risk for the development of lung cancer [176,177], with even passive smoking, or secondhand smoke, significantly increasing people’s risk [178,179]. Obese people have lower rates of smoking [180,181]. Smoking is an appetite suppressant due to the activating effects of nicotine on the hypothalamic pro-opiomelanocortin neuron pathway [182]. Nicotine does this by leading to α3β4 nicotinic acetylcholine receptor activation, which may contribute to smokers having lower BMIs [182,183,184]. Smokers tend to have greater abdominal obesity, suggesting that smoking may impact BMI and patients’ adipose tissue [185,186,187]. This increase in abdominal obesity is hypothesized to be secondary to increased cortisol levels, leading to downstream sequence such as insulin-resistant diabetes [188]. Therefore, using BMI, which does not have the sensitivity to detect abdominal obesity, we miss patients who are abdominally obese and whose weight is not enough to categorize them as generally obese [189,190].

## 4. Discussion

The obesity paradox complicates the prognostication of many oncologic diseases. This begs the question, why is there an obesity paradox? It is understood that obesity leads to cancer development through mechanisms such as immune dysfunction, but after a cancer diagnosis, obese patients tend to fair better. We believe one of the main drivers of the obesity paradox is the improper measurement of body habitus. By analyzing the multitude of ways body habitus may be measured, it is evident that BMI, though convenient, is severely limited. It is not flexible enough to account for variations secondary to lean body mass and different distributions of adiposity. A method such as VFI should be utilized to obtain a measurement that offers more granulated data for better comparisons and subsequent conclusions when analyzing a heterogeneous patient population. VFI also distinguishes the patterns of adipose storage for different patients and identifies central obesity, which is important for prognostication. Methods that rely on imaging modalities that allow slice analysis and densitometry analysis will also allow physicians to identify sarcopenia, which may be hidden in obese patients, leading to suboptimal outcomes.

When discussing the obesity paradox, the potentially confounding variables are numerous. Metformin use is more common in the obese population, as is statin usage. Both medications have been shown to confer a survival advantage when used post-diagnosis. Statin use has also been shown to improve long-term survival when patients are prescribed it prior to lung cancer diagnosis. These drugs may disproportionately benefit the overweight and obese population due to the increased usage in this population. An additional benefit of metformin usage is the protection from tobacco-derived carcinogens, which may partially explain why the obesity paradox is seen in lung cancer.

Another potential confounding variable is smoking status. Obese and overweight patients are less likely to be smokers. This may be because nicotine increases metabolism, and therefore the people who smoke are disposed to having lower BMIs. With the decreased prevalence of smoking in the obese population, they may have better pulmonary function and have an easier recovery or be better able to handle lung reduction surgery than their lower-BMI counterparts.

Finally, sex is an important confounding variable. It was shown that male patients benefit more from immunotherapy when used as monotherapy, but when combined with chemotherapy, female patients benefitted more, contributing to the obesity paradox.

Obesity leads to significant immunologic derangements, including upregulation of PD-1 on T cells. These contribute to oncologic pathogenesis and enhanced immune checkpoint inhibitor response when treated with anti-PD-1/PD-L1 inhibitors. Metabolic effects of obesity include deranged insulin signaling, increased steroid hormone signaling, increased glucose utilization, fatty acid utilization, and aberrant adipokine signaling [191]. Additional explanations include the differential effect that obesity has systemically versus locally at the tumor microenvironment (TME), a poignant example being the relative fatty acid starvation conditions present in the TME of obese patients.

## 5. Conclusions

In conclusion, the obesity paradox is highly nuanced and is affected by many confounding variables. BMI needs to be replaced with a measurement with a more thoughtful standardization process to account for the significant variability of body shapes and compositions. The immunologic and metabolic derangements that occur secondarily to obesity contribute to the obesity paradox. The interplay between statin, metformin, and cancer preferentially benefits those with higher BMIs, contributing to the obesity paradox in lung cancer. The decreased prevalence of smoking in obese patients as well as gender likely also play important roles in the obesity paradox. Further mechanistic studies will help us understand the obesity paradox better.

## Figures and Tables

**Table 1:** Published Literature.

Author	BMI Definition (kg/m^2^)	Number of Patients	Conclusions
Ferguson [104]	Overweight (25–29.9) Obese (30–34.9) Very obese (>35)	1369	Patients in the overweight, obese, and very obese categories had a lower rate of complications than patients with a BMI < 25 (OR: 0.72 *p* = 0.048)
Thomas [105]	Overweight (25–30) Obese (>30)	19,635	Overweight patients had lower mortality (OR: 0.72 *p* = 0.002) and obese patients had lower mortality (OR: 0.52 *p* < 0.001) compared to normal-weight patients and a statistically significant protective effect of obesity was observed in surgical complications
Williams [106]	Overweight (25–29.9) Obese I (30–34.9) Obese II (35–39.9) Obese III (>40)	41,466	Obese III patients had significantly higher rates of pulmonary complications (*p* < 0.001), but overweight, Obese I and Obese II patients had a lower risk of pulmonary complications and any post-operative event
Matsunaga [107]	Overweight (25–30) Obese (>30)	1518	Overweight and obese patients did not experience higher rates of pulmonary complications
Nakagawa [108]	Underweight (<18.5) Normal (18.5–25) Overweight (25–30) Obese (>30)	1311	Only underweight BMI was a poor prognostic factor for DFS (*p* = 0.03) OS (*p* = 0.03)
Lam [13]	Overweight (25–30) Obese (>30)	291	Increasing BMI was associated with improved survival (*p* = 0.011) and Obese individuals had a decrease of 31–58% (HR = 0.68 ± 0.21)
Attaran [110]	Obese (BMI > 30)	337	Survival rate was higher for obese patients (*p* = 0.02) on univariate analysis and (*p* = 0.04) on multivariate analysis
Petrella [115]	Obese (>25)	154	The high BMI group had a higher incidence of respiratory complications (*p* = 0.002) but there was no significant difference in ICU admission, LOS, and 30 day mortality
